# Transcription Factor AhR, Cytokines IL-6 and IL-22 in Subjects with and without Peri-Implantitis: A Case Control-Study

**DOI:** 10.3390/ijerph19127434

**Published:** 2022-06-17

**Authors:** Luis Ricardo Linard Martins, Kinga Grzech-Leśniak, Nidia Castro dos Santos, Lina J. Suárez, Gabriela Giro, Marta Ferreira Bastos, Jamil Awad Shibli

**Affiliations:** 1 Department of Periodontology, Dental Research Division, Guarulhos University, Guarulhos 07023-070, Brazil; ricardo.linard@hotmail.com (L.R.L.M.); nidia.castro@ymail.com (N.C.d.S.); lijsuarezlo@gmail.com (L.J.S.); 2 Laser Laboratory at Dental Surgery Department, Medical University of Wroclaw, 50-425 Wroclaw, Poland; kgl@periocare.pl; 3 Center for Clinical and Translational Research, The Forsyth Institute, Cambridge, MA 02142, USA; 4 Departamento de Ciencias Básicas y Medicina Oral, Universidad Nacional de Colombia, Cra 45 # 26-85, Bogota 11001, Colombia; 5 Programa de Pós Graduação em Ciências do Envelhecimento, Universidade São Judas Tadeu, Rua Taquari, 546, Sao Paulo 03166-000, Brazil; martafbastos@gmail.com

**Keywords:** gene expression, peri-implantitis, Th22 lymphocytes, transcription factor aryl-hydrocarbon receptor, interleukin-6, interleukin-22

## Abstract

Peri-implantitis is a plaque-associated condition characterized by mucosal inflammation and subsequent progressive loss of supporting bone; it is caused by bacterial biofilm, but the host response triggered by bacterial stimulation promotes the release of cells and mediators that culminate in tissue destruction. The Aryl-hydrocarbon Receptor (AhR) is associated with IL-22 production by Th22 and Th17 CD4+ Th cells. The presence of IL-6 may promote the Th22 phenotype. The present case-control study evaluated the gene expression of AhR, IL-22, and IL-6 in the peri-implant tissues of healthy and peri-implantitis patients. Tissue biopsies were collected from thirty-five volunteers (15 healthy and 20 with peri-implantitis). A real-time PCR reaction was utilized to assess the AhR, IL-22, and IL-6 gene expression levels relative to the reference gene (GAPDH). The results were analyzed using the Mann–Whitney test with a significance level of 5%. Higher levels of gene expression of AhR and IL-6 were detected in peri-implantitis tissues. The IL-22 gene expression levels did not differ between groups. In conclusion, higher gene expression levels for AhR and IL-6 were detected in the soft tissues of peri-implantitis patients. IL-22 did not vary between conditions, which may indicate the loss of the immunomodulatory role of IL-22 in periimplantitis.

## 1. Introduction

Dental implants have been used as a method for the rehabilitation of missing teeth, in which the long-term results reach notorious levels of success [1]. Despite well-established protocols, some failures can occur and lead to implant loss [2,3,4]. Dental implants, like teeth, can be colonized by bacteria [5]. The establishment of a submucosal dysbiotic process [6] causes an inflammatory response in the body that leads to alterations of the tissues that protect and support the implants [7], triggering clinical signs of illness. Two inflammatory diseases (mucositis and peri-implantitis) mediated by bacterial dysbiosis have been described around dental implants [8].

The knowledge of the factors that mediate the pathogenesis of peri-implantitis has recently evolved to the point of suggesting a division of the peri-implant conditions into subtypes according to the identified triggering factors [9,10]. Thus, confirming that there is an infectious origin in peri-implantitis, the pathogenesis of the disease in the context of the microbiome/host interrelation are beginning to be elucidated, as well as the role of microorganisms in the functioning of epithelial barriers immunity around implants.

For periodontitis, the dysregulation of the immune response has been described as an important part of tissue damage progression [11]. That regulation of immune response is given by cytokines with pro- and anti-inflammatory characteristics [12]. An imbalance in the production of cytokines can result in a destructive and progressive inflammatory response, thus determining the severity of the disease [13]. This may also play a fundamental role in the establishment and progression of peri-implant disease.

The aryl hydrocarbon receptor (AhR) is a latent, evolutionarily highly conserved cytoplasmic transcription factor whose expression is variable (and poorly characterized in many tissues) but which is highly expressed in epithelial barriers, especially in intestinal epithelial cells (IECs) and in cells of the gut-associated immune system (organ barriers) [14]. It is activated by low molecular weight molecules (barrier permeable) of different chemical nature, both xenobiotic and endogenous, which produces cell/ligand-specific transcriptomic changes, as well as changes in cell functions [15].

It has been documented that AhR could be involved in disease tolerance and may function as a sensor of bacterial danger [16]. There may be crosstalk (complex formation) with other proteins, including nuclear factor-kB (NFkB). AhR deficiency has been related to a decrease in IL-22 levels and therefore susceptibility to infections [17].

IL-22, which is a member of the Il-10 family, is recognized for its dual character, that is, protector and mediator of the pathogenesis of multiple infectious/inflammatory diseases [18,19]. As a protector agent it is part of the defense mechanisms against pathogens, as it acts on epithelial cells and induces their production of antimicrobial peptides [20,21], contributes to wound healing, and stimulates tissue regeneration [22,23]; on the other hand, as a mediator of pathogenic process it has been associated with various inflammatory diseases due to their association with osteoclastic differentiation and resorption activity (In vitro) [24].

Some non-lymphoid cells (fibroblasts, mast cells, macrophages, and neutrophils) can also produce IL-22 in different diseases [25,26], and innate lymphoid cells (ILCs), which reside at barrier surfaces, are also a main source of this cytokine [18].

It is also reported that AhR in the presence of immunological stimulation by proinflammatory cytokines or activation of Toll receptors regulates the expression of cytokine/chemokine genes, especially IL-6 [27], contributing to the function of the epithelial barrier.

The aim of the present case control study was to investigate the levels of gene expression of Ahr, IL-22, and IL-6 in the peri-implant soft tissues of peri-implantitis and healthy patients.

## 2. Materials and Methods

### 2.1. Study Population

This case-control study included partially or totally edentulous individuals who presented at least one implant-supported restoration in function for more than 2 years, as previously described [28]. This earlier study evaluated the levels of gene expression of the levels of RORγT and FOXP3 gene expression around healthy and diseased implants. Briefly, for inclusion in the study, the patients had to meet the following inclusion criteria: absence of lesions in the oral cavity, good oral hygiene, and indication of anti-infective surgical treatment for peri-implantitis (peri-implantitis group). Exclusion criteria were pregnancy or lactation; systemic diseases that could interfere with peri-implant tissues (osteoporosis, immune disorders, hepatitis, diabetes); use of systemic antibiotics 3 months prior to the sample collection; chronic use of medications that could interfere with immune-inflammatory response (e.g., corticosteroids, non-steroidal anti-inflammatories, immunosuppressive drugs, bisphosphonates) 3 months prior to the sample collection; chronic use of antimicrobial rinses (e.g., chlorhexidine, essential oils, cetylpyridinium chloride, triclosan). The experimental protocol was approved by the Research Ethics Committee of the University of Guarulhos (CAAE #0007.0.132.000-10) and the patients signed a free and informed consent form.

### 2.2. Clinical Parameters

Measurements of bleeding on probing (BoP), suppuration, probing depth (PD) in mm, and clinical attachment level (CAL) in mm were determined at six sites per implant for all the subjects included in the study. The PD and CAL measurements were recorded to the nearest mm using a North Carolina periodontal probe (PCPNU-15, Hu-Friedy, Chicago, IL, USA).

### 2.3. Peri-Implant Tissue Collection

The peri-implant tissue collection sites met the following criteria:

- Healthy group: dental implants scheduled to surgical procedures for non–disease-related reason procedures such as dental implant placement next to other implants, soft tissue grafting to modify peri-implant tissue phenotype.
- Peri-implantitis group: In order to obtain a biopsy of an area representative of the peri-implant inflammatory process, the mucosal tissue was removed around the implant with advanced peri-implantitis (PD ≥ 5 mm, bleeding on probing and/or suppuration, mobility and impairment of 2/3 of bone support). The tissue around a single implant was obtained from each individual with peri-implantitis.

### 2.4. Gene Expression Analysis

#### 2.4.1. RNA Extraction

Immediately after the biopsies were performed, the peri-implant mucosal tissue samples were packed in an RNAlater ^®^ solution (Ambion Inc., Austin, TX, USA) to prevent RNA degradation. Samples were incubated at 4 °C for 24 h and then stored at −20 °C until extraction. First, the RNA later solution was aspirated, and the tissue was packaged in liquid nitrogen for shredding. The triturated sample was then placed in TRIZOL reagent (Gibco BRL, Life Technologies, Rockville, MD, USA), homogenized for 30 s, and incubated for 5 min at room temperature. After this period, chloroform (Sigma, St. Louis, MO, USA) was added, and the samples were vortexed and centrifuged at 11,500 rpm for 15 min at 4 °C. The aqueous portion was transferred to another tube to which isopropanol was added, stirred, incubated for 20 min at −20 °C, and centrifuged as described above. RNA samples were subsequently resuspended in approximately 50 µL of diethylpyrocarbonate (DEPC) treated water and stored at −70 °C. Finally, the RNA concentration was determined by means of a spectrophotometer. Next, 1 µg of total RNA was evaluated for quality by 1% agarose gel electrophoresis.

#### 2.4.2. DNAse Treatment

Total RNA samples were treated for disposal of any DNA residue with DNAse (DNA-free TM, Ambion Inc., Austin, TX, USA) as recommended by the manufacturer. Buffer solution and DNAse turbo were added to the tubes with the extracted RNA, based on the previously evaluated RNA concentration. After shaking and centrifugation, the samples remained incubated at 37 °C for 30 min. Finally, the inactivator was added and the solution was stirred and centrifuged. Total RNA was again quantified by means of aspectrophotometer.

#### 2.4.3. Reverse Transcription

A total of 1 µg of the total DNA free RNA sample was used for cDNA synthesis. Reactions were performed to a final volume of 30 µL using the First-Strand cDNA Synthesis Kit (Roche Diagnostic Co., Indianapolis, IN, USA) following the manufacturer’s recommendations. Initially, the samples were incubated for 10 min at 25 °C and then for 60 min at 42 °C. After the second incubation step, the samples were incubated for 5 min at 95 °C and then for 5 min at 4 °C for cooling. The reagents used and their respective concentrations were buffer solution (1×), MgCl2 (5 mM), deoxynucleotides (1 mM), randomized primers (3.2 µg), RNAse inhibitor (50 U), and AMV reverse transcriptase (20 U).

### 2.5. Real-Time PCR (RT-PCR) Gene Expression Analysis

#### 2.5.1. Primer Design

The GAPDH (glycerin-aldehyd-3-phosphat-dehydrogenase, reference gene) primers for AhR, IL-22, and IL-6 were designed with the help of a program developed specifically for the preparation of primers for the LightCycler (Roche Diagnostics GmbH, Mannheim, Germany). All primers were checked for specificity by melting curve analysis, always using positive and negative controls. Table 1 shows the primer sequence, reaction profile, and amplicon size.

#### 2.5.2. Reaction Optimization

The efficiency for each gene was optimized before the start of the reactions. Concentrations ranging from 2.5 to 5 M for each pair of primers were used to determine under which conditions the reaction presented the best efficiency, as suggested by the equipment manufacturer, and 5 µM was chosen.

#### 2.5.3. RT-PCR Reactions

RT-PCR reactions were performed with the LightCycler system (Roche Diagnostics GmbH, Mannheim, Germany) using the FastStart DNA Master SYBR Green I kit (Roche Diagnostics GmbH, Mannheim, Germany). The reaction profile was determined following the protocol suggested by the equipment manufacturer. For each analysis, water was used as a negative control, and the reaction product was quantified using the manufacturer’s software (LightCycler Relative Quantification Software—Roche Diagnostics GmbH). GAPDH gene expression levels were used as reference (housekeeping) for normalization of values.

### 2.6. Statistical Analysis

Statistical analysis was performed using the Prism 7.0 software (GraphPad Software Inc., San Diego, CA, USA). Initially, the data were analyzed for normality using the Kolmogorov-Smirnov test and when the absence of normal values was detected, non-parametric statistical methods were used. Differences in gender frequency were assessed using Fisher’s exact test. Mean Student’s t-test evaluated age. All demographic data were presented as mean and standard deviation, except gender. The Mann–Whitney test performed comparisons of the levels of gene expression of the AhR transcription factor and the cytokines IL-6 and IL-22. The results were expressed as mean and standard deviation. The level of significance was set at 5% (*p* < 0.05).

## 3. Results

A total of 35 patients participated in this study, 16 females and 19 males. Initially, 35 samples were obtained and divided between the two experimental groups (healthy, *n* = 15, and peri-implantitis, *n* = 20). The periodontal parameters were collected for both groups and presented in the Table 2. Diseased implants presented more clinical inflammation when compared with non-diseased implants (*p* < 0.05).

During the RNA extraction processes, three samples (belonging to the peri-implantitis group) that did not have the necessary quality were excluded from proceeding with the analysis. In the real-time PCR step (RT-PCR), all samples showed expression of the reference gene (GAPDH). In total, 15 samples for the healthy group and 17 samples for the peri-implantitis group were included.

**Figure 1:**
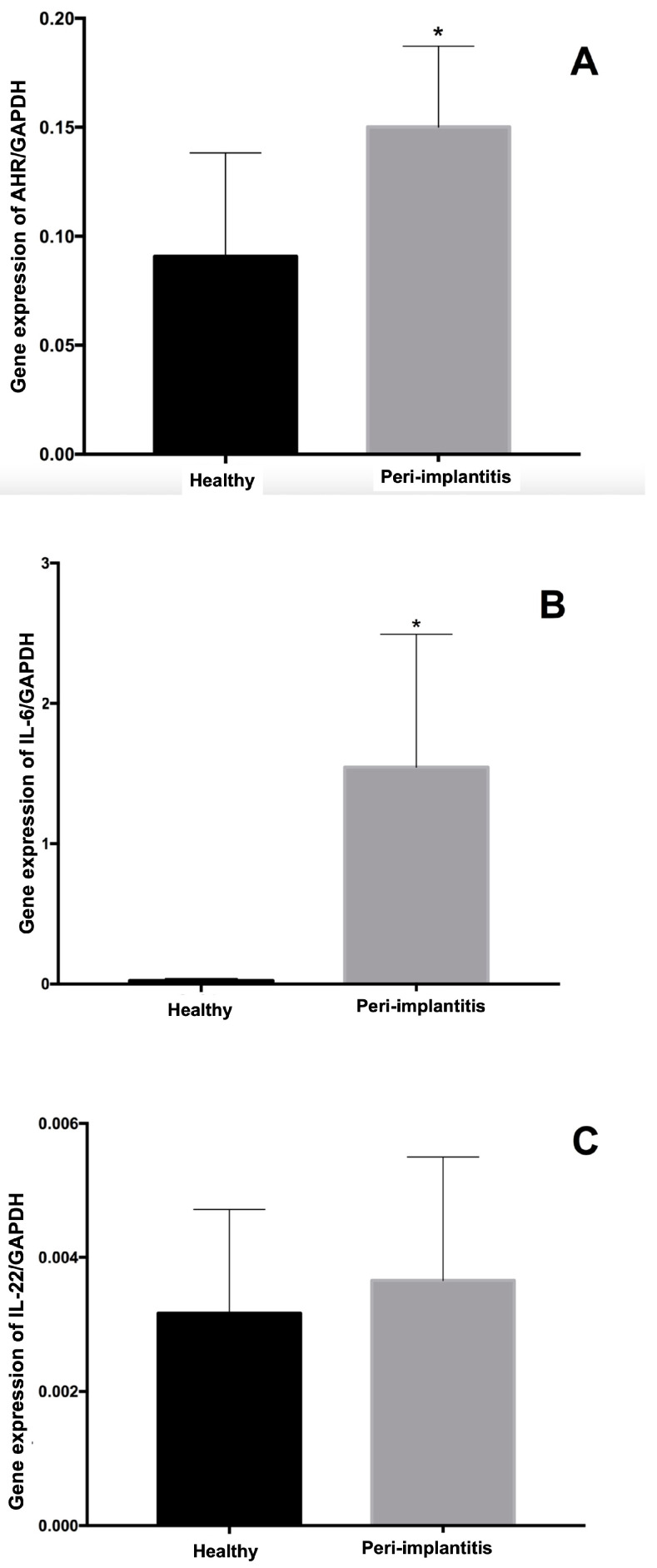
Gene expression levels of the transcription factor Aryl-hydrocarbon Receptor (AhR) (**A**), interleukin (IL)-6 (**B**), and IL-22 (**C**) according to the expression of the reference gene (GAPDH: glycerin-aldehyde-3-phosphate-dehydrogenase). * Statistically significant difference assessed using Mann–Whitney test (*p* < 0.05).

The results for the gene expression levels of the transcription factor AhR and the cytokines IL-6 and IL-22 regarding the reference gene GAPDH are shown in Figure 1. The highest levels of gene expression of the AhR-1 transcription factor and the IL-6 cytokine were found in the peri-implantitis group compared to the healthy group (*p* = 0.024 and *p* = 0.001, respectively). The analysis of IL-22 expression levels for the healthy and peri-implantitis groups did not reveal significant differences between groups (*p* = 0.46).

## 4. Discussion

Barrier organs such as mucosa from the oral–gut axis and skin, which are in continuous contact with external agents, including possible infectious agents, must be able to differentiate between physiological and pathological agents and subsequently activate immune responses according to the type of stimuli [29]. Mucosal tissues surrounding implants are no exception to this rule, and innate and adaptive host responses within the oral mucosa have been associated with the progression of peri-implant disease [30].

To evaluate possible alterations in the function of the epithelial barrier around implants that may be related to the occurrence of peri-implantitis, the gene expression of factors associated with the differentiation of Th cells in the Th22 subpopulation (AhR, IL-22, and IL-6) were evaluated in peri-implant soft tissues. Increased mRNA expression levels for Ahr and IL-6 were detected in diseased peri-implant tissues compared to healthy tissues. In addition, similar levels of IL-22 gene expression were observed in healthy and peri-implantitis patients, which shows a pattern contrary to what has been described for periodontal disease studying the Th22 T cells subpopulation [24,31].

The activators of AhR are, among others, natural substances found in yeasts and bacteria, stress factors and substances such as hydrogen and oxygen metabolites, metals, oxidized low-density lipoproteins, ozone, indoles, and even arachidonic acid metabolites such as lipoxin A4 [14]. This recognition of the microbiota and host-generated tryptophan metabolites has been proposed to explain the role of the AhR in innate immune signaling within barrier tissues in response to the presence of microorganisms.

Since peri-implantitis is an inflammatory disease initiated by bacteria, the changes in AhR expression and its activation may be directly related to the dysbiosis condition. A reciprocal interaction between the microbiota and the AhR has also been described where the microorganisms generate AhR activators, and consequently, the AhR-mediated host response regulates the microbiota through quorum-sensing activity [15]; this constitutes in itself a mechanism through which the epithelia try to control the changes in the microbiome in search of preventing the disease or its progression.

The responses activated by AhR may depend on the tissue environment, such as that generated by effector immune responses [32]. Epithelial barriers include multiple immune cells, many of which express differential levels of AhR: low levels in naïve T cells, helper T cells Th1 and Th2, and regulatory T cells, but high levels in Th17 cells and in both the interleukin (IL)-17/IL-22-producing and IL-17/IL-22-non-producing subsets of peripheral gd T cells. AhR is even recognized as a marker of the Th22 subpopulation of CD4+ T cells [33]. It is believed that Th17 cells that express ROR-γT are responsible for mediating the synthesis of AhR. This transcription factor, in turn, is necessary for the activation of cytokine production by some cell populations, such as the secretion of IL-22 by Th17 cells [34].

It is known that the mucosa acts as a protective physical barrier against the entry of microorganisms. In this context, it has been described that IL-22 is a cytokine produced by cells of the innate immune response, such as monocytes, dendritic cells, and natural killers. This cytokine has protective actions against the entry of bacteria and fungi, and it increases the proliferation of epithelial cells and the tissue repair [33,35,36,37,38]. IL-22 has distinct characteristics from other cytokines, as it is the only cytokine secreted by immune cells that acts primarily on non-immune epithelial cells with a unidirectional signaling flow [39].

Despite the protective functions described for IL-22, studies in a murine model of periodontitis progression establishing the presence of CD4+ AhR+ subpopulations that produce IL-22 in periodontal tissues reported a higher detection of these cells in periodontal lesions when compared with uninfected controls, and their association with alveolar bone loss [31].

Thus, it would be expected that in the presence of an infectious process, the activation of AhR would increase the production of IL-22 in search of modulating the inflammatory process and preventing further damage. In the present study, however, it was found that despite the increase in the gene expression of AhR, there was no significant increase in IL-22 gene expression pattern. Studies carried out to explain the host/microbiome relationship in other inflammatory conditions have found a reduction in the expression of IL-22 related to dysbiotic processes.

In a murine model of alcoholic liver disease, ethanol-associated dysbiosis reduced AhR activation levels, and thus intestinal Il-22 production was also decreased [40]. This could lead us to think of alterations in the regulation of inflammation mediated by Il-22 induced by dysbiotic changes that occur in peri-implantitis, which could be related to differences observed in the progression of periodontal and peri-implant diseases, where a faster progression pattern has been described for the infection around implants when compared to the infection around teeth [30].

The analysis of IL-6 gene expression in peri-implant tissues with peri-implantitis revealed the presence of increased levels of this cytokine compared to healthy tissues. In agreement with the data obtained in the present study, Severino et al. (2016) evaluated the levels of cytokines IL-6, IL-10, IL-17, and IL-33 in the peri-implant crevicular fluid by ELISA. Increased levels of IL-6 were detected compared to healthy peri-implant tissue [12]. In 2016, Duarte et al. conducted a systematic review of the cytokines involved in the pathogenesis of peri-implantitis. The analysis of 18 articles indicated moderate evidence of increased levels of pro-inflammatory cytokines, such as IL-6 [41]. More recently, Diaz-Zuniga et al. (2017) [42] conducted an in vitro study with monocyte-derived dendritic cells and CD4+ T cells from donors stimulated with *A. actinomycetemcomitans*. Increased levels of IL-6 produced by dendritic cells, and IL-22 and AhR by CD4+ T cells, were detected [42]. These results are partially in line with those found in the present study.

Finally, the cross-sectional study design does not allow a clear impact of the markers on the progression of peri-implant diseases. This limitation could impact on the gene expression markers and further prospective studies could clarify the role of transcription factor AhR in soft tissue sealing.

## 5. Conclusions

In conclusion, higher gene expression levels of the transcription factor AhR and the cytokine IL-6 were detected in the soft tissues of peri-implantitis patients, which can be interpreted as a reflection of the dysbiotic condition present around implants with peri-implantitis that exacerbated the inflammatory process. In peri-implant tissues, the low expression of IL-22 genes could imply an alteration in the modulation of the immune response at the level of the peri-implant mucosa.

This communication highlights the need for more studies on the role and modulation of IL-22 in peri-implant tissue loss.

## Figures and Tables

**Table 1:** Target genes, primers sequences, amplification profile, and amplicon size during Real Time PCR reaction AhR, Aryl-hydrocarbon Receptor; GADPH, glycerin-aldehyd-3-phosphat-dehydrogenase; IL, interleukin.

Gene	Sequence (5′–3′)	Amplification Profile [Temperature (°C)/Time (s)]	Amplicon Size (bp)
**AhR**	F: CAGTCTAATGCACGCCTG	95/10; 56/7; 72/7	155
	R: GTTGGTTGCCTCATACAACAC		
**IL-6**	F: CTGGCTTGTTCCTCACTAC	95/10; 56/7; 72/7	168
	R: GAACCTTCCAAAGATGGCTG		
**IL-22**	F: CTGATAACAACACAGACGTTCG	95/10; 56/7; 72/7	170
	R:CCACCTCCTGCATATAAGGC		
**GAPDH**	F: CTGAGTACGTCGTGGAGTC	95/10; 56/5; 72/10	250
	R: TGATGATCTTGAGGCTGTTGTC		

**Table 2:** Mean ± SD of the periodontal parameters and clinical characteristics of evaluated implant supported restoration from control (healthy, *n* = 15 subjects) and test (diseased, *n* = 20 subjects) groups. Mann–Whitney *U* test (* *p* < 0.05); ns: no significant.

	Health	Peri-Implantitis	*p* Value
PD (mm) *	3.42 ± 1.32	6.88 ± 0.11	**0.012**
CAL (mm) *	1.23 ± 0.54	5.58 ± 1.99	**0.023**
BoP (%) *	36.3 ± 13.3	80.8 ± 20.4	**0.005**
Suppuration (%) *	0 ± 0	19.3 ± 0.7	**0.001**
Time of loading (years)	5.2 ± 1.3	8.3 ± 2.4	*p* > 0.05
Maxilla:Mandible	6:9	8:12	*p* > 0.05

PD: Pocket Depth, CAL: Clinical Attachment Level, BoP: Bleeding on Probing.

## Data Availability

The data of this study can be released by email by the author responsible for correspondence upon request.
